# Concomitant Guillain-Barré syndrome and cerebral venous thrombosis complicating a SARS-CoV-2 infection: a case report

**DOI:** 10.11604/pamj.2022.42.212.31365

**Published:** 2022-07-19

**Authors:** Hajar Qorchi, Najib Kissani, Mohamed Chraa

**Affiliations:** 1 Neurology Department, University Teaching Hospital Mohammed VI, Marrakesh, Morocco,; 2 Laboratory of Clinical and Experimental Neuroscience, Faculty of Medicine, Cadi Ayyad University (UCAM), Marrakech, Morocco,; 3 Moroccan Society of Neurophysiology, Rabat, Morocco,; 4 Laboratory of Physiology, Faculty of Medicine and Pharmacy of Marrakech, Marrakech Morocco

**Keywords:** Cerebral venous thrombosis, Guillain-Barré syndrome, SARS-CoV-2, case report

## Abstract

Cerebral venous thrombosis associated to acute inflammatory axonal polyneuropathy during infection with SARS-CoV-2 (coronavirus-2) is unusual. We describe the case of a 66-year-old patient with typical clinical and electrophysiological criteria of acute axonal motor neuropathy, who was positive for SARS-CoV-2. The symptoms started with fever associated with respiratory symptoms, and complicated one week later by headaches, and general weakness. The examination showed bilateral peripheral facial palsy, predominantly proximal tetraparesis, and areflexia with tingling of limbs were found. The whole was concomitant with the diagnosis of an acute polyradiculoneuropathy. Electrophysiologic evaluation confirmed the diagnosis. Cerebrospinal fluid examination showed albuminocytologic dissociation, and brain imaging revealed sigmoid sinus thrombophlebitis. Neurological manifestations improved during treatment with plasma exchange and anticoagulants. Our case draws attention to the occurrence of cerebral venous thrombosis and Guillain-Barré syndrome (GBS) in patients with COVID-19. The neuro-inflammation induced by the systemic immune response to infection, can lead to neurological manifestations. Further studies should be conducted on the full clinical spectrum of patients with COVID-19 with neurological symptoms.

## Introduction

COVID-19 (coronavirus disease 2019), associated with SARS-CoV-2 (coronavirus-2), is initially described as a form of pneumonia, is now proven to be extremely variable in its manifestations, with systemic and multi-organ involvement, particularly at the cardiovascular and neurological levels. The neurological disorders fall into two categories depending on whether they affect the peripheral nervous system (PNS) or the central nervous system (CNS) [1,2].

Guillain-Barré syndrome is an acute, ascending and symmetrical polyradiculoneuritis, with sensory-motor impairment. Few cases are reported following infection with coronavirus (SARS-CoV-2). But in association of Guillain-Barré syndrome associated with cerebral thrombophlebitis has never been described to the best of our knowledge.

## Patient and observation

**Patient information:** a 66-year-old patient, with no past medical history, was admitted to our neurology department in September 2020 with bilateral peripheral facial palsy associated with acute proximally pronounced, symmetrical and synchronous tetraparesis, with acute headache.

**Clinical findings:** muscle testing was graded 3/5 proximally and 4/5 distally in the upper limbs, and 1/5 proximally and 2/5 distally in the lower limbs. Areflexia, numbness and tingling of all 4 limbs were also found. The whole was concomitant with the diagnosis of an acute polyradiculoneuropathy.

**Timeline:** he had fever, dry cough associated with vomiting one week before the onset of GBS symptoms. Given the existing epidemiological scenario and symptoms, COVID-19 infection was suspected.

**Figure 1:**
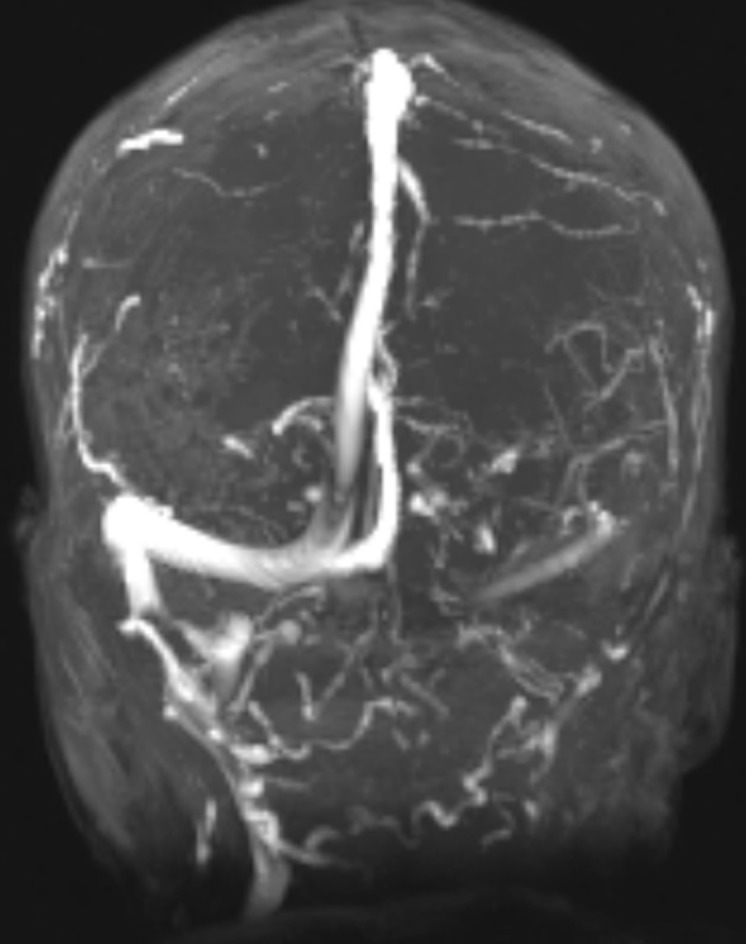
brain magnetic resonance imaging (MRI) in time-of-flight (TOF) sequence showing no visualization of the left sigmoid sinus

**Diagnostic assessment:** on arrival to our department, an electroneuromyography was done, showing reduced motor amplitude in all 4 limbs. Velocities were slightly reduced, and F waves were absent. Sensory nerve conduction studies were normal in all 4 limbs. Acute motor axonal polyradiculoneuropathy was diagnosed. Evaluation of cerebrospinal fluid (CSF) showed albuminocytologic dissociation with increased protein levels (1.38 g/L) and normal cell count. A polymerase chain reaction (PCR) test for COVID-19 resulted positive. He had in acute headache prompting cerebral magnetic resonance imaging (MRI) with angio-sequences that showed: a cerebral venous thrombosis of the left sigmoid sinus (Figure 1).

Standard laboratory tests (complete blood count, c-reactive protein (CRP), glycemia, creatinine, sodium and potassium levels, thyroid-stimulating hormone (TSH), creatine kinase and urine test) and serologies (HIV, hepatitis C virus (HVC), treponema pallidum hemagglutination/venereal disease research laboratory (TPHA/VDRL)) were also negative.

Our patient benefited from an exhaustive etiological assessment of cerebral venous thrombosis. The examination of the ear, nose, and throat (ENT) sphere was normal. Pathergic test was negative. The thrombophilia assessment is unremarkable, in particular no deficiency in protein C, S, and antithrombin III. The dosage of anticardiolipin, anti-b2 glycoprotein antibodies, and lupus anticoagulant was negative. Perinuclea anti-neutrophil cytoplamic (p ANCA), cytoplasmic anti-neutrophil cytoplasmic (c ANCA) antibodies, antinuclear antibodies (AAN), and angiotensin converting enzyme (ACE) were negative.

**Therapeutic intervention:** patient was started on hydroxychloroquine 200 mg dose three times per day for 10 days. He received 5 sessions of plasma exchange associated with low molecular weight heparin (LMWH) at a curative dose, with relay by vitamin K antagonist.

**Follow-up and outcomes:** the follow-up was marked by a considerable improvement in his respiratory and neurological state. His upper extremity weakness resolved after completion of the course of plasma exchange. Muscle testing was graded 4/5 proximally and 5/5 distally in the upper limbs, and 4/5 proximally and 5/5 distally in the lower limbs. A new polymerase chain reaction (PCR) test of an oropharyngeal sample was negative.

**Patient perspective:** in our case, the patient had a good clinical course with an improvement in symptoms. He was very satisfied with the treatment he received.

## Discussion

Neuro-inflammation may explain the neurological damage from COVID-19. SARS-CoV-2 is capable of causing hyper-inflammatory conditions secondary to a cytokine storm and elevation of interleukin-6 (IL-6). IL-6 has an important role in the failure of several organs. Excessive immunological reaction may be responsible for the major part of organ manifestations, including neurological complications [3].

The study by Lodigiani C *et al*. included 388 patients; thromboprophylaxis was used in 100% of intensive care unit (ICU) patients and 75% of those in the general wards. Thromboembolic events occurred in 21% of cases. Pulmonary embolism was confirmed in 10 patients. The rate of ischemic stroke and acute coronary syndrome was 2.5% and 1.1%, respectively [4]. According to Klok *et al*. pulmonary embolism was the most frequent thrombotic complication [5]. In the case we are reporting, it is a cerebral venous thrombosis.

Guillain-Barré syndrome is an acute, ascending and symmetrical polyradiculoneuritis, with sensory-motor impairment. Few cases are reported following infection with SARS-CoV-2 [6]. An Italian team has reported the onset of Guillain-Barré syndrome in 9 patients with COVID-19 [7]. The interval between the first symptoms of viral infection and those of Guillain-Barré was 5 to 10 days. The electrophysiology results were generally consistent with an axonal variant of Guillain-Barré syndrome in three patients and with demyelination in two patients. Scheidl *et al*. report the case of a 54-year-old patient with acute demyelinating inflammatory polyneuropathy (AIDP), who had PCR for SARS-CoV-2, 3 weeks before the onset of neurological symptoms. She had no respiratory or systemic symptoms, but anosmia and agnosia have been reported. It is treated with immunoglobulins with an improvement in neurological manifestations [8]. A Spanish team reported two cases of Miller-Fisher syndrome in COVID-19 patients, 3 to 5 days after the onset of flu-like symptoms. PCR was positive in an oropharyngeal sample, but negative in CSF. Two weeks later, both patients achieved complete neurological recovery [9]. The clinical and electrophysiological results of our patient were consistent with acute motor axonal neuropathy (AMAN), like the previous cases. But the association of Guillain-Barré syndrome with cerebral thrombophlebitis has never been described to the best of our knowledge.

## Conclusion

An immune response to COVID-19 can lead to neurological manifestations. Further studies should be conducted on the clinical spectrum of patients with COVID-19 with neurological involvement.

## References

[1] Garg RK (2020). Spectrum of neurological manifestations in COVID-19: a review. Neurol India.

[2] Helms J, Kremer S, Merdji H, Clere-Jehl R, Schenck M, Kummerlen C (2020). Neurologic features in severe SARS-CoV-2 infection. N Engl J Med.

[3] Flis-Richard H, Verdonk F (2020). Atteintes neurologiques dans l´infection au SARS-CoV-2 (COVID-19). Prat Anesth Reanim.

[4] Lodigiani C, Iapichino G, Carenzo L, Cecconi M, Ferrazzi P, Sebastian T (2020). Venous and arterial thromboembolic complications in COVID-19 patients admitted to an academic hospital in Milan, Italy. Thromb Res.

[5] Klok FA, Kruip MJHA, van der Meer NJM, Arbous MS, Gommers DAMPJ, Kant KM (2020). Incidence of thrombotic complications in critically ill ICU patients with COVID-19. Thromb Res.

[6] Khatoon F, Prasad K, Kumar V (2020). Neurological manifestations of COVID-19: available evidences and a new paradigm. J Neurovirol.

[7] Toscano G, Palmerini F, Ravaglia S, Ruiz L, Invernizzi P, Cuzzoni MG (2020). Guillain-Barré syndrome associated with SARS-CoV-2. N Engl J Med.

[8] Scheidl E, Canseco DD, Hadji-Naumov A, Bereznai B (2020). Guillain-Barré syndrome during SARS-CoV-2 pandemic: a case report and review of recent literature. J Peripher Nerv Syst.

[9] Gutiérrez-Ortiz C, Méndez-Guerrero A, Rodrigo-Rey S, San Pedro-Murillo E, Bermejo-Guerrero L, Gordo-Mañas R (2020). Miller-Fisher syndrome and polyneuritis cranialis in COVID-1 Neurology.

